# *Heracleum persicum* Essential Oil Nanoemulsion: A Nanocarrier System for the Delivery of Promising Anticancer and Antioxidant Bioactive Agents

**DOI:** 10.3390/antiox11050831

**Published:** 2022-04-25

**Authors:** Shima Ghareh Bashlouei, Ehsan Karimi, Mohsen Zareian, Ehsan Oskoueian, Majid Shakeri

**Affiliations:** 1 Department of Biology, Mashhad Branch, Islamic Azad University, Mashhad 917568, Iran; shimagharabashlou@gmail.com; 2 Department of Biology and Biological Engineering, Chalmers University of Technology, 41296 Göteborg, Sweden; mohsen.zareian@chalmers.se; 3 Department of Research and Development, Arka Industrial Cluster, Mashhad 9188944586, Iran; 4 Department of Medicine, University of Washington, Seattle, WA 98109, USA

**Keywords:** phytochemicals, medicinal plants, plant-based nanostructures, eco-friendly nanotherapeutics, volatile compounds, therapeutics, phytobiotics, apiaceae family

## Abstract

Essential oils are important compounds for the prevention and/or treatment of various diseases in which solubility and bio-accessibility can be improved by nanoemulsion systems. *Heracleum persicum* oil nanoemulsion (HAE-NE) was prepared and biological properties were investigated against human breast cancer cells and normal human fibroblasts foreskin. Particle size, zeta potential and poly dispersity index were 153 nm, −47.9 mV and 0.35, respectively. (E)anethole (57.9%), terpinolene (13.8%), ɣ-terpinene (8.1%), myrcene (6.8%), hexyl butyrate (5.2%), octyl butanoate (4.5%) and octyl acetate (3.7%) was detected in nanoemulsion. Proliferation of cancer cells at IC_50_ = 2.32 μg/mL was significantly (*p* < 0.05) inhibited, and cell migration occurred at 1.5 μL/mL. The HAE-NE at 1.5, 2.5 and 3.5 µg/concentration up-regulated caspase 3 and enhanced sub-G1 peak of cell cycle with nil cytotoxic effects in the liver, kidney and jejunum of mice. Villus height, villus width, crypt depth and goblet cells in mice group fed with 10 and 20 mg/kg body weight of HAE-NE improved. Cellular redox state in the liver indicated 10 and 20 mg/kg body weight of nanoemulsion significantly up-regulated the expression of SOD, CAT and GPx genes. *Heracleum persicum* oil nanoemulsion could be an eco-friendly nanotherapeutic option for pharmaceutical, cosmetological and food applications.

## 1. Introduction

*Heracleum persicum*, a flowering plant of the family Apiaceae, naturally grows under humid conditions and has traditionally been used as a medicinal herb and flavoring agent. The potential of *H. persicum* for use in biomedicine, food and pharmaceutical industries has recently attracted much attention [1,2]. *Heracleum persicum* has been extensively used as food additives, food preservatives, flavoring agents and spices (i.e., to flavor pickles) and for the treatment purposes of gastrointestinal, neurological, respiratory, urinary and rheumatologically dysfunctions [1,3]. Physiological functions of *H. persicum* can be antioxidant, antihyperlipidemic, antidiabetic, anti-inflammatory, anticonvulsant, antimicrobial, analgesic and cardio- and gastro-protective properties [4,5,6,7]. The major bioactive compounds identified to induce such physiological effects are volatiles, ethyl esters, n-alkenes, phenolics, flavonoids, alkaloids, terpenoids and triterpenes [1]. Hexyl butyrate, anethole, octyl acetate, hexyl-2-methylbutanoate and hexyl isobutyrate were found to be the main natural phytochemicals of *H. persicum* essential oil [1,7].

In traditional medicine, essential oils are known to act as pharmaceutical agents, and thereby, can be used for the prevention and/or treatment of various diseases [8]. A wide spectrum of volatile bioactive phytochemicals, e.g., phenolics, flavonoids and terpenes, can be found in the essential oils of plant origins [9,10]. The solubility and bio-accessibility of essential oils can be improved by nanoemulsion systems, which ultimately can be developed as drug delivery systems [11,12]. Nanoemulsions are nanoscaled emulsion-based systems containing therapeutic compounds intended to keep pharmaceutical ingredients active and deliver components to the target cells in a more effective fashion [13]. Nanoemulsions are typically made of a stable isotropic system containing two immiscible liquid types, which are basically mixed together to form stable nanodroplets with a 20–200 nm range [13]. Characteristics and details of various nanoemulsion systems were broadly described earlier [13,14] and will not be discussed here. Recently, nanoscale emulsion systems have gained much interest and attention—thanks to special characteristics, i.e., high performance and stability [15]. As such, nanoemulsions continue to be developed as one of the main formulation systems in the pharmaceutical and cosmeceutical industries [15]. The present study, therefore aimed to synthesize a nanoscale emulsion system from the essential oils of *Heracleum persicum*. Cellular and molecular characteristics of the nanoemulsion against cancer cell line, cytotoxicity and antioxidant properties in vitro and in vivo were also investigated. To the best of our knowledge, this is the first report of a nanoscale emulsion system made from the Persian herbal medicine *Heracleum persicum* for future development in the pharmaceutical and cosmeceutical industries.

## 2. Materials and Methods

### 2.1. Chemicals, Reagents and Cell Lines

Polysorbate 20 and 80, Tween 80 (polyethylene glycol sorbitan monooleate), polyethylene glycol (PEG), DMEM culture medium trypsin, MTT [3-(4, 5-dimethylthiazol-2-yl)-2, 5-diphenyltetrazolium bromide] and Fetal Bovine Serum (FBS), were purchased from (Merck, Darmstadt, Germany). Cancer cell lines (MDA-MB-231, ATCC HTB 26) and human fibroblasts foreskin (ATCC SCRC-1041) as normal cell line were obtained from the Pasteur Institute of Iran. The PCR Master Mix and SYBR Green Master mix were purchased from Vazyme Biotech (Nanjing, China). The RNA extraction kit and cDNA Synthesis Kit were purchased from CinnaGene (Tehran, Iran).

### 2.2. Essential Oil Extraction Procedure

The essential oils in the aerial parts of *H. persicum* were extracted by a Soxhlet apparatus. In brief, 200 g dried *H. persicum* was mix with 1000 mL ethanol and boiled for 2 h, after which the extracted solution was separated by a vacuum controller as previously described [16].

### 2.3. Nanoemulsion Preparation, Identification and Characterization

Nanoemulsion was prepared as described previously [17] with slight modifications. Briefly, 3 g of *H. persicum* oil extract were mixed with 97 mL deionized water and sonicated for 30 min at 20 kHz ultrasonic frequency. The ratio of essential oil to non-ionic surfactant was 1:3 *v/v*. The HAE-NE characteristics, i.e., size, polydispersity index (PDI) and dynamic light scattering (DLS), was also measured. The Z-average values and morphology of nanodroplets were analyzed according to the distribution intensity and field emission scanning electron microscopy (FESEM), respectively. Volatile bioactive compounds present in HAE-NE were detected by Gas Chromatography-Mass Spectrometry as previously described [18]. One microgram of the oil sample was injected into GC (Agilent 6890) coupled with an Agilent 5973N Mass Spectrometer (Santa Clara, CA, USA). Essential oils were analyzed on a BPX5 fused silica column (30 m × 0.25 mm i.d. × 0.25 μm) under electronic impact mode (70 eV), split injection ratio (1:35) and He as carrier gas (initial pressure: 110 kPa), with 0.5 mL/min flow rate under a scan range of 40–500 amu. The temperature program of the oven was set as follows. Initially, 50 °C for 5 min, then a gradient of 3 °C/min up to 240 °C, after which it was raised at 15 °C/min up to 300 °C and finally held at 300 °C for 3 min. Compounds were identified by comparing retention time (RT) with the literature and mass spectra library, i.e., the National Institute of Standards and Technology (NIST).

### 2.4. Anticancer Assay

The anticancer effects of HAE-NE were assessed against human breast cancer cell line (MDA-MB-231), whereas human fibroblasts foreskin (HFF) was used as normal cell line [19]. Briefly, 5 × 10^3^ cells were seeded for 24 h after which a varying concentration of HAE-NE (0.7, 1.5, 3.1 and 6.2 µg/mL) were treated against cell lines. After 48 h incubation, the medium was refreshed with the media containing MTT (0.5 mg/mL) incubated for 3 h at 37 °C, after which the medium was substituted with the media containing dimethylsulfoxide (DMSO, 100 µL). Optical density (OD) of the samples were recorded at 570 nm and cell viability was calculated as follows:

Cell viability (%) = (OD Sample/OD Control) × 100

### 2.5. Cell Migration Assay

The impact of the HAE-NE on the migration ability of human breast cancer cell line was conducted in vitro as previously described [19]. In brief, cells were first cultured to reach 80–100% confluency and a series of lines were made using a sterile 10-µL micropipette tip. Medium was replaced and washed completely with new medium to remove detached cells. The wounds were photographed (0 h) and the medium containing 0 and 1.5 µg/mL HAE-NE were added, after which cells were incubated at 37 °C under 5% CO_2_ for 10 h and photos of the wounds were taken. The migration distance was calculated by deducting the distance of wound edges at 0 and 10 h.

### 2.6. Gene Expression of Caspase 3

The profiling of the caspase 3 was evaluated using the Real-Time PCR as previously described [20]. The RNA was extracted at the end of the cell incubation using RNeasy Mini Kit (Qiagen, Hilden, Germany) and cDNA was synthesized using cDNA synthesis kit (Qiagen, Hilden, Germany) as per the manufacturer’s protocols. The PCR reaction conditions were optimized and set as follows: 94 °C for 5 min (1×), 94 °C for 25 s, 58 °C for 30 s and 72 °C for 25 s (40×). All RT-PCR amplifications were performed in triplicate and primers are listed in Table 1.

### 2.7. Flow Cytometry-Based Assay

Various concentrations of HAE-NE (0.7, 1.5, 3.1, and 6.2 µg/mL) were treated against the cancer cell line for 48 h, after which cells were washed and mixed with Propidium Iodide (PI), 0.2% Triton X-100 and 0.1% sodium citrate and maintained for 10 min at 37 °C under dark conditions as previously described [20]. The AO/PI dyes were mixed with the harvested and washed cells for staining purposes and were observed under a fluorescence microscope (Olympus BX41, Tokyo, Japan). A laser flow cytometer (FACSCalibur, Becton Dickinson, TX, USA) was also performed to determine the cell cycle alterations.

### 2.8. In Vivo Assay

The toxicity of HAE-NE was determined using 15 female mice (22–25 g) kept in individual cages (24 °C with 50% humidity) and maintained for 1 week to adapt to the laboratory condition. Animals were randomly divided into three groups (*n* = 5) and treated as follows: a control group were orally gavaged with distilled water, whereas the other two groups orally received HAE-NE at 10 and 20 mg/kg body weight, respectively. The experiment lasted for 30 days. All animal experiments were conducted according to the ethical principles approved by Azad University ethics codes (IR.IAU.MSHD.REC.1399.141).

### 2.9. Histopathological Analysis and Tissue Staining

For histopathological analyses and tissue staining, animals, at the end of animal trials, were first euthanized and the liver, kidney and jejunum were obtained and washed with 0.9% NaCl serum, maintained in formalin 10%, after which paraffinized and cut into thin pieces (5 μm) [21]. Tissue samples were stained with hematoxylin and eosin as previously described [17,22]. Cell morphologies were investigated using an inverted microscope and results were interpreted by an experienced pathologist for further tissue characterization.

### 2.10. Antioxidant Gene Expression in Mice Liver

The expressions of SOD, CAT and GPx, which are basically recognized to be important antioxidant biomarkers, were investigated in mice liver. The effect of different concentration of nanoemulsion (0, 10 and 20 mg/kg body weight) were investigated as previously described [23]. In brief, the liver tissues of the mice were crushed and prepared for RNA extraction using the RNeasy Mini kit (Qiagen, Hilden, Germany), after which a Quantitect Reverse Transcription Kit (Qiagen, Hilden, Germany) was employed for the synthesis of cDNA libraries and primer sequences targeted for CAT, SOD and GPx genes and a house keeping (GAPDH) gene were also designed (Table 1). To perform a comparative Real-Time PCR, a SYBR Green PCR Master Mix (Qiagen, Hilden, Germany) was employed and designated genes were amplified under the following program: 95 °C for 5 min (1×), 95 °C for 20 sec, 55 °C for 20 sec and 72 °C for 25 sec (35×). The gene expressions were normalized to GAPDH as a reference gene, after which they were normalized to the expression of the respective genes in the control group.

### 2.11. Statistical Analyses

Data were reported as mean ± standard deviation. All experiments were performed in triplicate. The statistical analyses were carried out using one-way ANOVA by the statistical package SPSS (version 21, SPSS Inc., Chicago, IL, USA) and α at 95% was defined as a statistically significant difference. Symbols on figures indicated differences between groups—ns: *p* ≥ 0.05, *: *p* < 0.05, **: *p* < 0.01 and ***: *p* < 0.001.

## 3. Results and Discussion

### 3.1. HAE-NE Characterization and Identification

**Figure 1:**
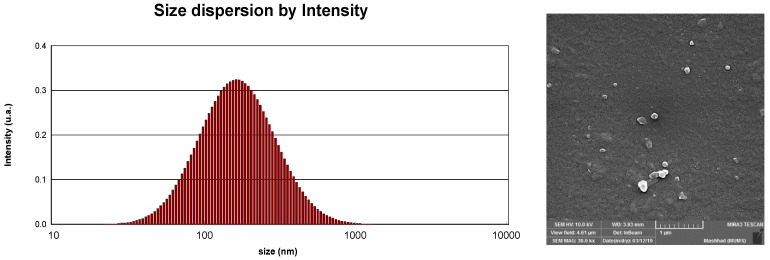
The characteristics of *Heracleum persicum* essential oil nanoemulsion and anticancer effect of HAE-NE. The dynamic light scattering results revealed that the nanoemulsion size dispersion was 153.64 nm (**left**). The Field Emission Scanning Electron Microscopy indicated the spherical shape of HAE-NE, which was in accordance with particle size data (**right**).

The particle size, poly dispersity index and zeta potential of HAE-NE were 153.64 nm, 0.35 ± 0.07 and −47.9 mV, respectively. The poly dispersity index in our study (0.35 ± 0.07) is, in principle, less than 0.3, which indicates homogenous dispersion [24]. The zeta potential is also an index which demonstrates the stability of colloidal dispersions. A zeta potential value outside −30 to +30 mV is typically recognized as satisfactory repulsive force to achieve and maintain physical colloidal stability [24]. The dynamic light scattering results revealed that the nanoemulsion size dispersion was 153.64 nm (Figure 1, left). The Field Emission Scanning Electron Microscopy indicated the spherical shape of HAE-NE, which was in accordance with particle size data (Figure 1, right).

Bioactive compounds were also detected by GC-MS in HAE-NE, in which the main volatile compounds were anethole (57.9%) and terpinolene (13.8%) (Table 2). Anethole was also identified as major compound in the oil composition of leaf (47.5%) and flower (38.6%) of *H. persicum* [25]. Similar volatiles identified in our study (Table 2) were previously reported to be present in various parts of *Heracleum persicum* [25,26,27,28]. Variation in the percentage of volatiles in the essential oil content within various *Heracleum persicum* species is mostly influenced by environmental factors [27]. The HAE-NE in our study (Table 2) also contained hexyl butyrate (5.2%), octyl butanoate (4.5%) and octyl acetate (3.7%). Essential oils rich in octyl acetate and hexyl butyrate are valuable for medicinal and commercial purposes [27,28]. Other bioactive compounds identified in HAE-NE (Table 2) were reported to exhibit a variety of biological properties, e.g., bactericidal, antioxidant and anti-inflammatory activities [7,28,29,30,31].

**Figure 2:**
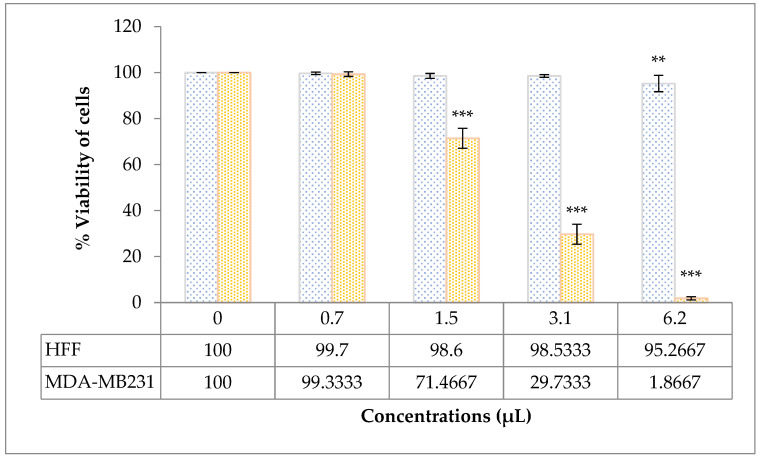
Effect of *Heracleum persicum* oil nanoemulsion (HAE-NE) on human fibroblasts foreskin (HFF) and human breast cancer cell (MDA-MB-231) viability. Values represent the mean ± standard deviation from three independent experiments. Symbols provided on bars indicated differences between groups—no symbol: *p* ≥ 0.05, **: *p* < 0.01 and ***: *p* < 0.001.

The cytotoxicity potential of HAE-NE against human cancer cell line (MDA-MB-231) and normal cell line (HDF) indicated that HAE-NE inhibited the cancer cell proliferation significantly (*p* < 0.05) with IC_50_ = 2.32 μg/mL (Figure 2). Furthermore, various concentrations of HAE-NE did not show toxicity affects against the normal cell (HFF). Similar findings on the effect of *H. persicum* essential oils against human cancer lines of HeLa, LS180, were also reported [28]; however, the IC_50_ in the present work was found to be lower (Figure 2) than previous reports.

### 3.2. Migration Analysis

**Figure 3:**
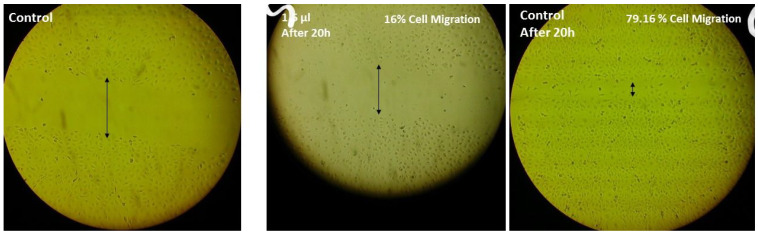
Migration inhibition percentage of HAE-NE in MDA-MB231 cells after 20 h of incubation. Error bar indicates the standard error of the mean of three independent experiments. Cells were visualized at 200× magnification.

The potential of HAE-NE on MDA-MB231 cell migration at the concentration of 1.5 μL/mL incubation indicated the untreated MDA-MB231 cells demonstrated complete wound closure after 20 h (Figure 3). The HAE-NE at 1.5 μL/mL concentration exhibited strong inhibition on the migration of breast cancer cells. Cell migration is deemed to be important in cancer progression to incurable metastatic disease [32]. Therapeutics which potentially block the migration of cancer cells may inhibit/reduce metastasis, and thereby, can profoundly improve cancer therapies [33]. Such functionality is of particular interest and prime importance in cancer treatments such as triple negative breast cancer, in which targeted drugs are currently not reasonably developed [33].

### 3.3. In Vitro Gene Expression Profiling of Caspase 3

**Figure 4:**
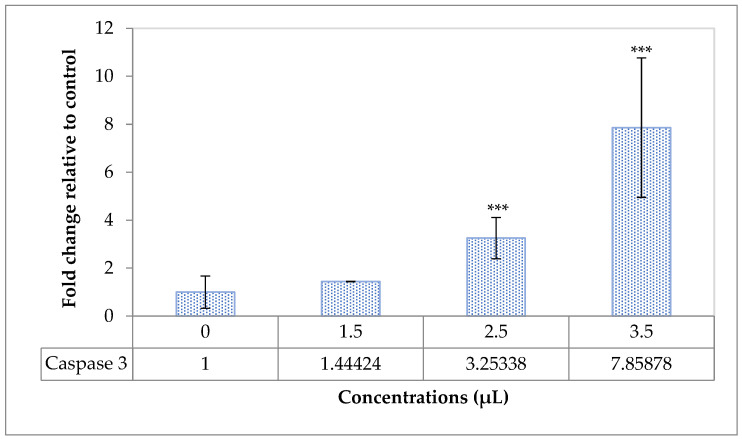
The expression of caspase-3 gene in human breast cancer cell line (MDA-MB231) with different concentrations of *Heracleum persicum* oil nanoemulsion (HAE-NE), indicating the HAE-NE can induce apoptosis death in breast cancer cell line. Symbols provided on bars indicate differences between groups—***: *p* < 0.001.

Different concentration of HAE-NE (1.5, 2.5 and 3.5 µg/mL) significantly (*p* < 0.05) up-regulated Caspase 3 gene (Figure 4), indicating the HAE-NE can induce apoptosis death in breast cancer cell line. Caspase-3—a cysteine-aspartic protease—is known to play a key role in apoptotic pathways, which basically interferes with the apoptosis response. The expression of Caspase-3 is typically influenced by the status of oxidative stress and antioxidant defense systems. The overexpression of Caspase-3 may ultimately induce apoptosis responses. Essential oils present in plant extracts can remarkably induce Caspase-3-dependent apoptosis properties and examples of such plant extracts include, but are not limited to, *Afrostyrax lepidophyllus* Mildbr, *Monodora myristica*, *Arachis hypogaea*, *Ricinus communis* L. *Garcinia epunctata* and *Ptycholobium contortum* [34,35,36,37].

### 3.4. Flow Cytometry Analysis

**Figure 5:**
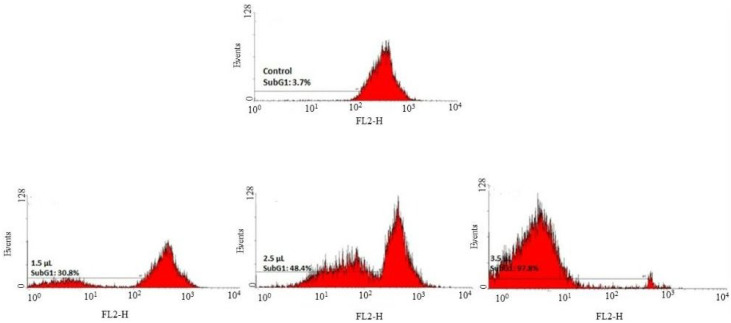
Flow cytometric analysis of cell cycle in human breast cancer cells (MDA-MB-231) upon treatment with different concentrations of *Heracleum persicum* oil nanoemulsion (HAE-NE) during 48 h. Increase in HAE-NE concentration enhanced the sub G1 peak of the cell cycle in MDA-MB-231 cells, thereby confirming cell apoptotic death, which could be recognized as a potential apoptosis inducer.

The flow cytometry data showed the increase in HAE-NE concentration and enhanced sub-G1 peak of the cell cycle in MDA-MB-231 cells, and confirmed cell apoptotic death, which could be recognized as a potential apoptosis inducer (Figure 5). Similar to our findings, essential oils present in lemongrass (*Cymbopogon citratus* Stapf) were also reported [38] to induce cell cycle arrest in cancer cells. Similarly phytochemicals of *Bulbine frutescens* demonstrated cell cycle arrest at G1 phase and induced apoptosis in breast cancer cells [39].

### 3.5. Histopathological Alterations and Morphometric Analysis

**Figure 6:**
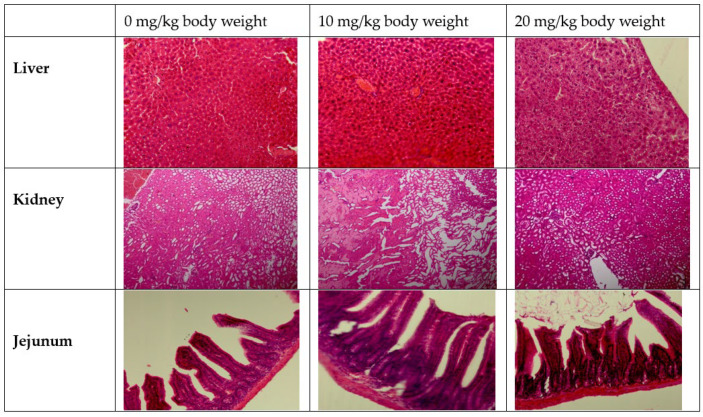
Histopathological analysis of live, kidney and jejunum of the mice treated with different concentration of *Heracleum persicum* oil nanoemulsion. Treatment with *Heracleum persicum* oil nanoemulsion (HAE-NE) showed that cellular death was not detected in tissues.

The histopathological features of the liver, kidney and jejunum tissue upon treatment with HAE-NE show changes in the histomorphology and that cellular death were not detected in tissues (Figure 6), and the morphometric analyses such as villus height, villus width, crypt depth and goblet cells (Table 3) were in accordance with the findings of the jejunum histopathology. The mice group fed 10 and 20 mg/kg body weight of HAE-NE also showed an enhancement in the morphometric parameters. Goblet cells secrete mucin to protect the internal wall of the intestine and HAE-NE in our study increased the number of goblet cells (Table 3).

### 3.6. Antioxidant Gene Profiling In Vitro

The profiling of antioxidant genes, i.e., SOD, CAT and GPx, as the cellular redox state in liver indicates 10 and 20 mg/kg body weight of nanoemulsion significantly (*p* < 0.05) up-regulated the expression of antioxidant-related genes (Table 4). Similar antioxidant capacities of *H. persicum* aqueous extract in diabetes-induced oxidative stress rats were also reported [6], in which antioxidant defense systems in the brain, kidney and liver were enhanced upon supplementation with plant extract or antioxidants [40]. Another observation made in a human trial with minimal coronary artery disease confirmed antioxidant effects of *H. persicum* fruit extract in modulating oxidative stress biomarkers, such as SOD, CAT and GPx activities [41]. Such antioxidant properties were mainly attributed to (E)-anethole present in *H. persicum* extracts [28]. Cancer cells tend to counteract the effects of free radicals via antioxidant enzymes, such as SOD, CAT and GPx as a mechanism to increase cell viability under stressful conditions caused by augmented metabolism.

In the light of these research, it may be concluded that *H. Persicum* Essential Oil Nanoemulsion could considered as a promising antioxidant and anticancer natural drug in breast cancer therapy. Further research on the various phytochemicals of individual groups of natural components may demonstrated the exact capacity of *H. Persicum* Essential Oil to inhibit different cancer cells and encourage the improvement as novel broad-spectrum herbal antioxidant and anticancer formulation in future.

## 4. Conclusions

The present study demonstrated preparation and characterization of *H. persicum* oil nanoemulsion with biological properties in vitro and in vivo. Bioactive compounds present in the nanoemulsion were also detected. In addition to nanoemultion properties against normal and cancer cell lines, the cytotoxic effects in the liver, kidney and jejunum of mice and the enhancement of the morphometric parameters were also demonstrated and discussed. Given the several side effects of synthetic drugs for the treatment purposes of intestinal diseases which could ultimately deteriorate patients’ conditions, *Heracleum persicum* oil nanoemulsion, in our study, exhibited the potentials to be developed as therapeutic drugs. Our finding suggested *H. persicum* oil nanoemulsion could be an eco-friendly nanotherapeutic option for pharmaceutical, cosmetological and food application purposes.

## Figures and Tables

**Table 1:** Details of the primer sets used in the study.

	Gene	Forward (5′→3′)	Reverse (5′→3′)	Accession Number
MDA-MB231 cell line	Caspase-3	CTGGACTGTGGCATTGAGAC	ACAAAGCGACTGGATGAACC	NM_001284409
	GAPDH ^1^	GAAGGTGAAGGTCGGAGTC	GAAGATGGTGATGGGATTTC	NM002046
Mice tissue	SOD	GAGACCTGGGCAATGTGACT	GTTTACTGCGCAATCCCAAT	NM_011434
	CAT	ACATGGTCTGGGACTTCTGG	CAAGTTTTTGATGCCCTGGT	NM_009804
	GPx	CACAGTCCACCGTGTATGCC	GTGTCCGAACTGATTGCACG	NM_008160
	GAPDH	GACTTCAACAGCAACTCCCAC	TCCACCACCCTGTTGCTGTA	NM_001289726

^1^ GAPDH: glyceraldehyde 3-phosphate dehydrogenase; SOD: superoxide dismutase; CAT: catalase; GPx: glutathione peroxidase.

**Table 2:** Major compounds present in the nanoemulsion synthesized using essential oil obtained from aerial parts of *H. persicum*.

Peak	Compound	Percentage
1	(E)-anethole	57.9
2	terpinolene	13.8
3	ɣ-terpinene	8.1
4	myrcene	6.8
5	hexyl butyrate	5.2
6	octyl butanoate	4.5
7	octyl acetate	3.7

**Table 3:** Morpho-characteristics of mice jejunum treated with different concentration of *Heracleum persicum*.

	0 mg/kg Body Weight	10 mg/kg Body Weight	20 mg/kg Body Weight	SEM
Villus Height (µm)	322	341	362	3.89
Villus Width (µm)	68	76	94	4.38
Crypt Depth (µm)	71	76	75	5.94
Goblet Cells	2.3	3.1	3.8	0.29

SEM: Standard Error of the Mean. Means with different superscript letters indicate significant difference at *p* < 0.05.

**Table 4:** The expression analyses of antioxidant-related genes in the liver of mice.

	0 mg/kg Body Weight	10 mg/kg Body Weight	20 mg/kg Body Weight	SEM ^1^
SOD ^2^	1 ^c^	1.9 ^b^	2.8 ^a^	0.06
CAT	1 ^c^	1.6 ^b^	2.3 ^a^	0.08
GPx	1 ^c^	1.5 ^b^	1.9 ^a^	0.05

^1^ SEM: Standard Error of the Mean. ^2^ SOD: Superoxide dismutase; CAT: Catalase; GPx: Glutathione peroxidase. Means with different superscript letters indicate significant difference at *p* < 0.05.

## Data Availability

Data is contained within the article.
